# Evaluation of Antifungal Activity of Chromenoquinoline Derivatives Synthesized in the Medium Temperature Water Using Isinglass–Bassorin Cryogel as a Sustainable Catalyst

**DOI:** 10.1002/open.70297

**Published:** 2026-09-14

**Authors:** Shahrzad Abdolmohammadi, Behrooz Mirza, Sina Azadi, Zinatossadat Hossaini

**Affiliations:** ^1^ Department of Chemistry ST.C. Islamic Azad University Tehran Iran; ^2^ Department of Chemistry Ka.C. Islamic Azad University Karaj Iran; ^3^ Department of Chemistry SR.C. Islamic Azad University Tehran Iran; ^4^ Department of Chemistry QaS.C. Islamic Azad University Qaemshahr Iran

**Keywords:** antifungal, aqueous medium, catalyst recyclability, chromenoquinoline derivatives, green synthesis, isinglass–bassorin cryogel

## Abstract

Our team subscribes to using contemporary, ecologically sound, and sustainable catalysts to produce a range of chromenoquinolines. This was accomplished utilizing the environmentally friendly heterogeneous catalyst isinglass‐bassorin (IG‐BR) cryogel in aqueous media at room temperature in a one‐pot three‐component reaction involving 4‐hydroxycoumarin, aromatic aldehydes, and 3,4‐(methylenedioxy)aniline. The two main significant advantages of this innovative and practical method are the use of water as an ecologically friendly medium and the IG‐BR cryogel as a strong and recyclable heterogeneous catalyst (for at least 4 cycles) that conforms with green chemistry principles to produce target compounds in a brief reaction time (4–5 h) and high product yields (89%–93%). Additionally, all target compounds demonstrated superior inhibition zones at doses of 50 and 75 μg/mL when tested for antifungal activity in vitro against three types of fungal pathogens in comparison with two common antifungal medications.

## Graphical abstract

In this work, a number of chromenoquinolines were synthesized in an aqueous medium at room temperature using isinglass–bassorin (IG‐BR) cryogel as an eco‐friendly heterogeneous catalyst. Furthermore, when tested for antifungal efficacy in vitro against three different kinds of fungal infections, all target compounds showed better inhibitory zones than two widely used antifungal drugs.

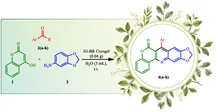

## 1 Introduction

According to recent research, porous materials with strong clinical translation and outstanding liquid adsorption capabilities have a lot of promise for use in both the food industry and medical devices [1]. As members of the porous materials family, cryogels, as low‐cost, chemically stable, biocompatible, and nontoxic materials, are unique hydrogels generated in mildly frozen solutions of monomeric or polymeric precursors. Due to their high porosity, persistent pores, rapid hydration, and well‐designed structure, these materials are considered ideal candidates for adsorbents in various applications, including tissue engineering [2], bioactive delivery [3], food preservation [4], and environmental remediation, where they serve as promising catalysts or adsorbents [5, 6, 7, 8]. The mechanical extrusion impact of ice crystal development, during the process, drives a highly linked three‐dimensional network design by multidentate hydrogen bonding connections [9]. This dual effect concurrently accomplishes the material's advantageous mechanical characteristics and through‐pore structure in addition to increasing the sequential physical and chemical interlinking density [10, 11]. Compared with other porous materials, cryogels are extensively utilized in food preservation, packaging, and transportation to shield against contamination and external damage due to their larger porosity and specific surface area [12].

According to the literature study, the quinoline motif is one of the well‐known chemical classes with key importance in the field of chemistry [13]. Diverse pharmacological applications of quinoline and its derivatives are explored thanks to their broad range of biological activities, such as antibacterial [14], antiviral [15], antifungal [16], anticancer [17], and antituberculosis [18]. Researchers have also reported their effectiveness in treating high cholesterol [19], rheumatoid arthritis [20], and Alzheimer's disease (AD) [21].

Recently published studies on green synthetic approaches for synthesizing heterocyclic scaffolds have mostly focused on the heterogeneous approach [22, 23, 24, 25, 26, 27]. Also, green methods for organic synthesis appreciate water as a useful reaction medium because it easily fits into different conditions, helping to support sustainable and environmentally friendly chemical processes [28].

To treat fungal infections, which often manifest on the nails, hair, and skin, antifungal care is an essential treatment class. For this reason, the synthesis of novel compounds with antifungal potential is of interest to medicinal chemists in the fight against fungal illnesses [29, 30, 31].

**SCHEME 1:**
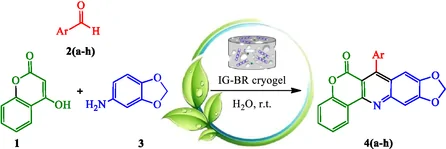
| Synthesis of chromenoquinolines in water by IG‐BR cryogel as catalyst.

So far, our research group has published many green and sustainable synthetic methodologies utilizing the efficacy of novel heterogeneous catalysts [32, 33, 34, 35, 36, 37, 38]. The main goal of this research is to expand a water‐mediated isinglass‐bassorin cryogel‐catalyzed synthesis for the preparation of a series of chromenoquinoline derivatives. For this purpose, first, the IG‐BR cryogel composite as a new heterogeneous catalyst will be easily obtained through a very simple and cost‐effective pathway using the natural products as the raw materials. Then, it plays the role of an efficient novel heterogeneous catalyst in a three‐component cyclocondensation reaction of 4‐hydroxycoumarin, aromatic aldehydes, and 3,4‐(methylenedioxy)aniline to produce chromenoquinolines (**4a‐h**) in aqueous media at room temperature (Scheme 1).

## 2 Experimental Section

### 2.1 Materials and Methods

All reagents and solvents were purchased from Merck and Fluka and used without further purification. We took the melting points with an Electrothermal 9100 apparatus and recorded the IR spectra on an ABB FTLA 2000FT‐IR spectrometer and acquired the ^1^H NMR and ^13^C NMR spectra on a Bruker DRX‐500 AVANCE at 500 MHz and 125 MHz, respectively, in deuterated dimethyl sulfoxide (DMSO‐d6) as the solvent with tetramethylsilane (TMS) as the internal standard. We also performed the elemental analyses measurements on the Foss–Heraeus CHN‐O‐rapid analyzer. The powder X‐ray diffraction (XRD) pattern of the catalyst was obtained on a Rigaku Ultima IV diffractometer with X’Pert PRO diffractometer using Cu Kα (λ = 1.54 Å) radiation. The field‐emission scanning electron microscopy (FESEM) of the catalyst was revealed using a scanning electron microscope (SEM, Tescan‐Mira 4). Nitrogen adsorption–desorption experiments were carried out at 77 K on a Belsorpmini II accelerated surface area and porosimetry system. The Brunauer–Emmett–Teller (BET) surface area (SBET) was calculated based on the linearity of the BET equation.

### 2.2 General Procedure for the Synthesis of IG‐BR Cryogel

**FIGURE 1:**
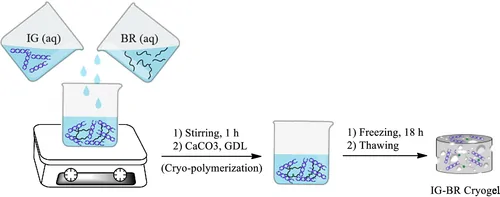
Preparation process of IG‐BR composite cryogel.

The IG‐BR cryogel was prepared according to a previously reported method [39] with slight modifications. Initially, the BR and IG starting materials were obtained from the thermal analysis (TG) [40] and commercial isinglass [41], respectively, and separately dispersed in deionized water to obtain 1% (w/v) BR solution and 1% (w/v) IG solution. Each mixture was subjected to constant stirring for 1 h and then mixed together. To induce gelation, a 1.5% (w/v) CaCO_3_ solution was subsequently added to the above mixture and dispersed evenly throughout the system. The glucono‐δ‐lactone (GDL) powder was then added to achieve a 3.5% (w/v) concentration, followed by uninterrupted stirring until achieving complete dissolution and homogeneous distribution. The mixture was kept at ambient temperature for 6 h, which allowed the IG‐BR cryogel to form completely. After formation, the cryogel was washed thoroughly with deionized water. The prepared cryogel was first frozen at −20 °C for 12 h. They were then thawed at ambient temperature for 12 h to produce the IG‐BR composite cryogel. The preparation procedure for the IG‐BR composite cryogel was shown in Figure 1.

### 2.3 General Procedure for the Preparation of Compounds 4a‐h

A mixture of 4‐hydroxycoumarin (**1**, 1 mmol), aromatic aldehyde **2** (1 mmol), 3,4‐(methylenedioxy)aniline (**3**, 1 mmol), and IG‐BR cryogel (0.04 g) was suspended in distilled water (5 mL) and stirred at room temperature for an appropriate duration (Table 1) with assessed using TLC. Upon reactions’ completion, the mixture was diluted with 0.5 mL N,N‐dimethyl formamide (DMF), and the catalyst was recycled from the reaction mixture by centrifugation followed by washing and drying for several hours at room temperature. Thereafter, the solid product was crystallized from aqueous DMF to give the pure product.

**TABLE 1:** IG‐BR cryogel catalyzed synthesis of chromeno[*b*][1,3]dioxolo[*g*]quinolinones 4a‐h.

Products	Ar	**Yield, %** a ^,^ b	Time, h	M.p, °C	
				Obs.	Lit
**4a**	C_6_H_5_	89	5	245–247	246–247 [42]
**4b**	4‐Br‐C_6_H_4_	92	4.5	279–281	284–285 [42]
**4c**	4‐Cl‐C_6_H_3_	91	4	249–251	249–251 [42]
**4d**	4‐NC‐C_6_H_4_	93	4	260–262	260–262 [42]
**4e**	3‐HO‐C_6_H_4_	90	5	282–283	280–281 [42]
**4f**	4‐CH_3_O‐C_6_H_4_	89	4.5	274–275	274–275 [42]
**4g**	2‐CH_3_‐C_6_H_4_	91	5	217–219	221–222 [42]
**4h**	3‐O_2_N‐C_6_H_4_	92	5	216–217	211–213 [42]

a Yields refer to those of pure isolated products.

b Reaction conditions: a mixture of 4‐hydroxycoumarin (**1**, 1 mmol), aromatic aldehyde **2** (1 mmol), 3,4‐(methylenedioxy)aniline (**3**, 1 mmol), and IG‐BR cryogel composite (0.04 g) in H_2_O (5 mL) was stirred at room temperature for 4–5 h.

#### 2.3.1 7‐Phenyl‐6H‐chromeno[4,3‐b][1,3]dioxolo[4,5‐g]quinoline‐6‐one (4a)

White solid, yield: 0.327 g (89%), m*p* = 245–247 °C (lit: 246–247 C [42]). IR (KBr): ν_max_ 3075, 2727, 2610, 1669, 1609, 1560, 1348, 1310, 1271, 1211, 1100 cm^−1^. ^1^H NMR (500 MHz, DMSO‐*d*_6_): 6.03 (1H, d, *J* = 1.1 Hz, H‐10), 6.12 (1H, d, *J* = 1.1 Hz, H‐10´), 7.17 (3H, br t, *J* = 8.5 Hz, H_Ar_), 7.30 (2H, d, *J* = 8.3 Hz, H_Ar_), 7.33 (2H, br t, *J* = 7.6 Hz, H_Ar_), 7.36 (1H, br s, H_Ar_), 7.38 (1H, br s, H_Ar_), 7.60 (1H, br t, *J* = 7.7 Hz, H_Ar_), 7.92 (1H, br d, *J* = 7.6 Hz, H_Ar_) ppm. ^13^C NMR (125 MHz, DMSO‐*d*_6_): *δ* 100.1, 103.6, 108.0, 115.5, 117.2, 122.9, 123.2, 125.1, 126.2, 127.6, 128.6, 128.9, 131.4, 134.1, 139.2, 141.1, 150.0, 153.7, 164.3, 164.6, 169.7 ppm. Anal. Calcd. For C_23_H_13_NO_4_ (367.36): C, 75.20; H, 3.57; N, 3.81. Found: C, 75.07; H, 3.47; N, 3.97.

### 2.4 Antifungal Susceptibility Testing

Using the conventional disk diffusion method (Kirby–Bauer test), the products’ inhibitory efficacy against a few chosen fungal pathogens was assessed [43]. Pathogenic fungi were grown on Mueller Hinton agar plates (Scharlau Microbiology, Spain). A swab containing a 1.5 × 10^6^ CFU/mL fungus suspension was used to inoculate the plates. The suspension was diluted using a sterile 0.9% sodium chloride solution. After that, the compounds tested and the conventional antifungal agents, including nystatin or terbinafine as the positive control, were separately dissolved in ethanol using ultrasonic sonication. Sterile filter paper discs (6 mm in diameter) were impregnated with the respective solutions of compounds tested and standard antifungal drugs at concentrations of 50 and 75 μg/mL, respectively. The plates were incubated at 37 °C for the whole night. After 18 h of incubation, the diameter of the inhibition zone surrounding each disk was measured in order to document the growth inhibition. All of the aforementioned tests were carried out in triplicate to guarantee the repeatability of the observations.

### 2.5 Result and Discussions

**FIGURE 2:**
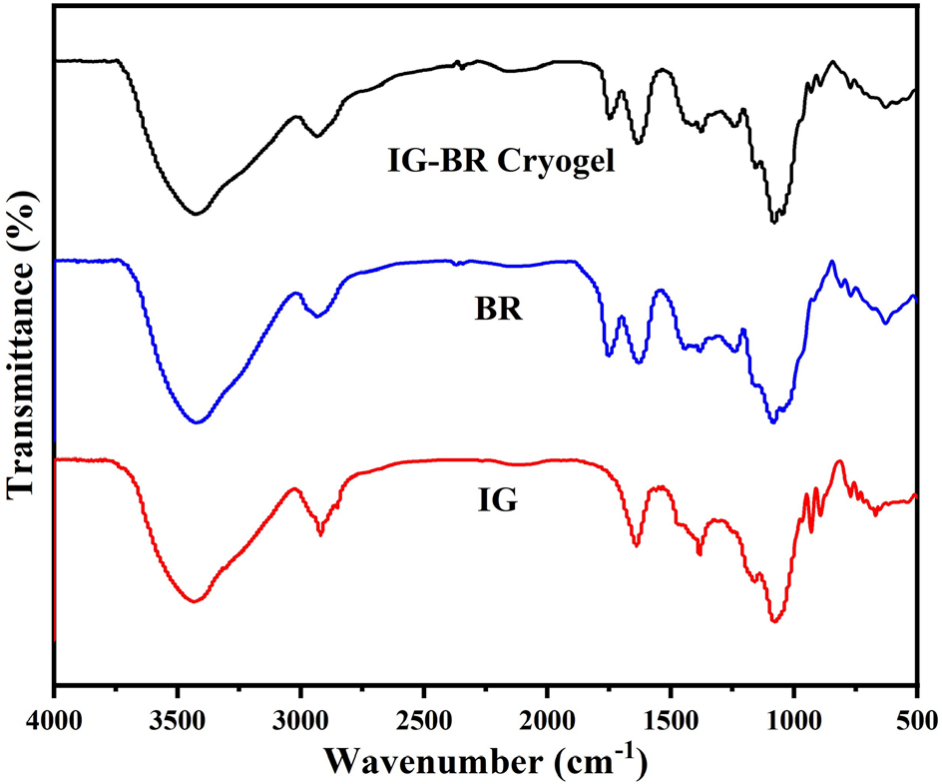
FT‐IR spectrum of the IG‐BR composite cryogel.

First, the previously described approach [39] was used to produce the IG‐BR composite cryogel. The structure and purity of the produced IG‐BR composite cryogel were characterized using Brunauer–Emmett–Teller (BET), X‐ray diffraction (XRD), field emission scanning electron microscopy (FESEM), and Fourier transform infrared spectroscopy (FT‐IR). Figure 2 displays the synthesized composite cryogel's FT‐IR. The stretching vibration of the hydroxyl group (OH) of the BR, intramolecular hydrogen bonds, and adsorbed water are represented by the peaks seen at 3200–3600 cm^−1^. The C—H stretching of BR and IG compounds is responsible for the adsorption peaks seen in the 2850–2920 cm^−1^ range. The carboxyl and amide groups’ C=O stretching vibrations are responsible for the adsorption maxima at 1750 and 1640, respectively. Furthermore, the C—O stretching vibration of carboxylate groups is linked to the distinctive peak at 1110 cm^−1^.

**FIGURE 3:**
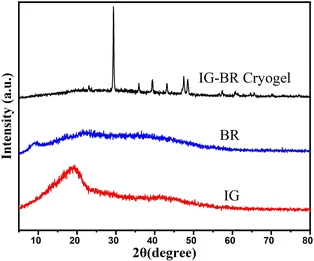
Powder XRD pattern of the IG‐BR composite cryogel.

Figure 3 illustrates the XRD pattern of the synthesized composite cryogel. The strong interactions between the two polymer components that reduce the composite's crystallinity are confirmed by the absence or displacement of the respective diffraction peaks of IG and BR from their original locations in the IG‐BR composite cryogel [44]. Moreover, the distinct reflection peaks observed at 2*θ* = 21.8°, 26.5°, 29.4°, 35.9°, 39.4°, 43.0°, 47.5°, and 48.4° are likely attributable to residual CaCO_3_ and GDL.

**FIGURE 4:**
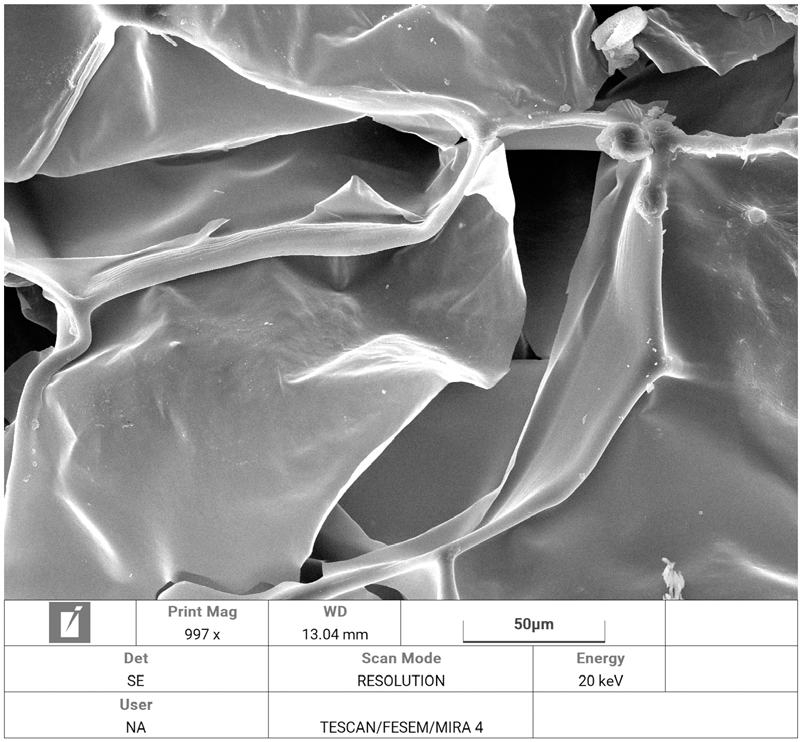
SEM image of the IG‐BR composite cryogel.

Figure 4 shows the composite's SEM picture, which provides comprehensive details on the IG‐BR cryogel's surface condition and shape. A 3D, extremely porous structure typical of cryogel with random orientation created by the ice templating process is shown by the SEM analysis of the composite. At various magnifications, the linked network of fibers and pores is visible.

**FIGURE 5:**
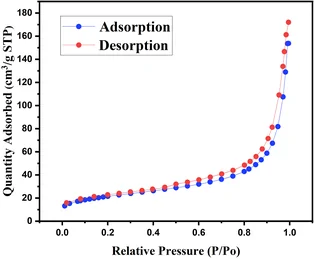
BET image of the IG‐BR composite cryogel.

The BET spectrum of the IG‐BR composite cryogel is displayed in Figure 5. A type IV nitrogen sorption isotherm is demonstrated by the composite's BET surface area and nitrogen adsorption/desorption isotherm data, confirming the synthesized mesoporous composite's consistent pore diameter with multilayer physisorption. The composite's BET and BJH analyses verified the IG‐BR composite cryogel's pore surface area of 74.29 m^2^ g^−1^, mean pore diameter of 14.51 nm, and total volume of 0.27 cm^3^ g^−1^.

**SCHEME 2:**
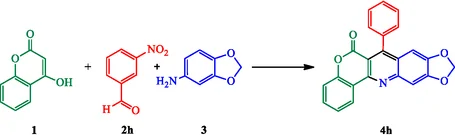
| Synthesis of 7‐(3‐nitrophenyl)‐6*H*‐chromeno[4,3‐*b*][1,3]dioxolo[4,5‐*g*]quinoline‐6‐one (**4h**) under various conditions.

We took the reaction of 4‐hydroxycoumarin (**1**), 3‐nitrobenzaldehyde (**2h**), and 3,4‐(methylenedioxy)aniline (**3**) as the template reaction to screen the amount of catalyst, the appropriate temperature, and the reaction environment conditions (Scheme 2 and Table 2). First, we investigated the IG‐BR cryogel's effectiveness in relation to both catalyst‐free conditions and a few typical acid or basic catalysts. Under catalyst‐free conditions at reflux water, it was found that the reaction proceeds slowly and yields little (Table 2, entry 1). When compared with some commonly studied catalysts and their various concentrations, IG‐BR cryogel performs best when employed at a concentration of 0.04 g (Table 2, entries 2–6). In the next stage of optimization, the refluxing temperature with 0.04 g of catalyst in water was also tested. It was shown that conducting reflux conditions is just relatively faster without significant variation in the yield of the product (Table 2, entry 6 vs. entry 8). Furthermore, control experiments with other organic solvents (namely, CH_3_CN, CH_2_Cl_2_, DMF, and EtOH) showed that aqueous media was better suited for the target reaction (Table 2, entry 6 vs. entries 9–12). Finally, the ideal optimum conditions were determined to be aqueous‐mediated conditions at room temperature with 0.04 g of catalyst. Such reaction conditions can offer a mild, safe, and environmentally friendly path.

**TABLE 2:** Optimization for three‐component reaction between of 4‐hydroxycoumarin (1), 3‐nitrobenzaldehyde (2h), and 3,4‐(methylenedioxy)aniline (3).

Entry	Catalyst	Reaction condition	Time, h	Yield, %^a^
1	None	(H_2_O, 10 mL), reflux	12	32
2	HCl (5 drops)	(H_2_O, 10 mL), r.t.	5	50
3	*p*‐TsOH (0.02 g)	(H_2_O, 10 mL), r.t.	5	62
4	DAHP (0.02 g)	(H_2_O, 10 mL), r.t.	5	68
5	IG‐BR cryogel (0.03 g)	(H_2_O, 10 mL), r.t.	5	80
6	IG‐BR cryogel (0.04 g)	(H_2_O, 10 mL), r.t.	5	92
7	IG‐BR cryogel (0.05 g)	(H_2_O, 10 mL), r.t.	4	92
8	IG‐BR cryogel (0.04 g)	(H_2_O, 10 mL), reflux	2	94
9	IG‐BR cryogel (0.04 g)	(CH_3_CN, 10 mL), r.t.	5	73
10	IG‐BR cryogel (0.04 g)	(CH_2_Cl_2_, 10 mL), r.t.	5	79
11	IG‐BR cryogel (0.04 g)	(DMF, 10 mL), r.t.	5	75
12	IG‐BR cryogel (0.04 g)	(EtOH, 10 mL), r.t.	5	80

Abbreviations: DAHP, diammonium hydrogen phosphate; DMF, dimethylformamide; *p‐*TsOH, *p‐t*oluenesulfonic acid.

a Isolated yield.

Once the conditions were tuned, a variety of substituted benzaldehydes were used to prepare chromeno[*b*][1,3]dioxolo[*g*]quinolinones, which improved the protocol's efficiency (Table 1). The structures of the produced compounds were verified using elemental analysis and spectroscopic techniques (IR and NMR).

One of the principal benefits associated with heterogeneous catalysts is their capacity for recovery and reuse, which directly affects the economics and sustainability of catalytic processes. The model reaction under ideal conditions and on a large scale was used to assess the reusability of the IG‐BR cryogel catalyst. As seen in Table 3, the catalyst maintained its high catalytic efficiency for at least four consecutive cycles with just a little yield loss.

**TABLE 3:** Evaluation of reusability of catalyst for the synthesis of 5h.

Run	1	2	3	4	5
Time, h	1	1	1	1	1
Yield, %^a^^,^ b	92	91	91	89	86

a Isolated yield.

b Reaction conditions: a mixture of 4‐hydroxycoumarin (**1**, 25 mmol), 3‐nitrobenzaldehyde (**2h**, 25 mmol), 3,4‐(methylenedioxy)aniline (**3**, 25 mmol), and IG‐BR cryogel composite (1 g) in H_2_O (5 mL) was stirred at room temperature for 5 h.

**SCHEME 3:**
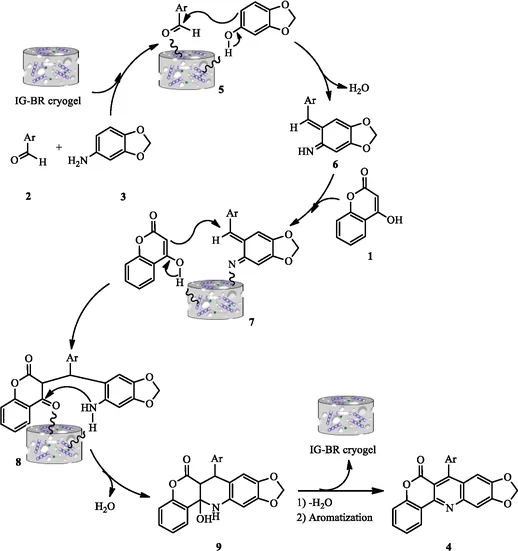
| Proposed mechanism for the synthesis of compound **4**.

Highlighting the essential role of IG‐BR cryogel active sites, it is suggested that the composite cryogel, which involves acidic sites and hydroxyl (—OH) groups, typically can activate the carbonyl group of aromatic aldehyde **2** via acid‐base interactions. In order to produce alkene **6**, it was then suggested that this intermediate be subjected to a direct Knoevenagel reaction with 3,4‐(methylenedioxy)aniline (**3**). Intermediate **8** is produced by the subsequent Michael‐type addition of 4‐hydroxycoumarin (**1**) to alkene **6**. Intermediate **8** is cyclized, dehydrated, and aromatized to produce the end product **4**. The proposed mechanism is illustrated in Scheme 3.

The comparison of the current method with the previously published process for synthesizing 7‐(3‐nitrophenyl)‐6*H*‐chromeno[4,3‐*b*][1,3]dioxolo[4,5‐*g*]quinoline‐6‐one highlights an advancement in utilizing milder conditions. Additionally, this comparison reinforces the case for expanding the use of IG‐BR cryogel composite as a highly efficient green catalyst for the sustainable synthesis of bioactive heterocyclic scaffolds (Table 4).

**TABLE 4:** Comparison of the catalytic efficiency of the IG‐BR cryogel composite with previously reported catalyst for the preparation of 7‐(3‐nitrophenyl)‐6*H*‐chromeno[4,3‐*b*][1,3]dioxolo[4,5‐*g*]quinoline‐6‐one (4h).

Conditions	**Yield, %** a	References
4‐Hydroxycoumarin (1 mmol), 3‐nitrobenzaldehyde (1 mmol), 3,4‐(methylenedioxy)aniline (1 mmol), and Cu/ZnO@GO nanocomposite (0.05 g) in H_2_O (3 mL), under sonication for 15 min with the power of 80 W, at room temperature	96	[42]
4‐Hydroxycoumarin (1 mmol), 3‐nitrobenzaldehyde (1 mmol), 3,4‐(methylenedioxy)aniline (1 mmol), and IG‐BR cryogel composite (0.04 g) in H_2_O (5 mL), under stirring for 5 h at room temperature	92	This work

a Isolated yield.

### 2.6 Antifungal Evaluation

For the antifungal impact experiments, three phytopathogenic fungi were chosen: Epidermophyton floccosum (*E*. *floccosum*), Microsporum canis (*M*. *canis*), and Trichophyton mentagrophytes (*T*. *mentagrophytes*). The antifungal efficacy of the produced compounds against the aforementioned bacterial strains at doses of 50 and 75 μg/mL was assessed using the disk diffusion technique. Table 5 provides a summary of the findings. All of the compounds were shown to have moderate to high inhibitory effects against the three dermatophyte isolates listed, when compared with two common antifungal medications.

**TABLE 5:** Antifungal effectiveness of the biogenic synthesized compounds against the tested phytopathogenic fungi.

Compounds	Conc., μg/mL	**Inhibition diameter, mm** a		
		*E*. *floccosum*	*M*. *canis*	*T*. *mentagrophytes*
**4a**	50	17	21	22
	75	19	24	24
**4b**	50	20	18	21
	75	22	20	22
**4c**	50	16	19	24
	75	18	21	26
**4d**	50	17	23	22
	75	21	25	23
**4e**	50	22	22	21
	75	20	24	24
**4f**	50	18	23	23
	75	20	24	25
**4g**	50	20	22	21
	75	21	23	24
**4h**	50	23	20	24
	75	25	22	26
Nystatin	50	19	20	23
	75	22	22	24
Terbinafine	50	24	25	26
	75	26	26	28

a 1–10 mm = resistant; 11–14 mm = intermediate; ≥15 mm = sensitive.

## 3 Conclusion

In summary, IG‐BR cryogel was prepared as a new recyclable heterogeneous catalyst through a simple procedure using nature's starting materials. The presented catalyst was applied to the preparation of chromenoquinoline derivatives under environmentally friendly and mild conditions. The structures of target products were confirmed using techniques such as melting point, FT‐IR, ^1^HNMR, and ^13^C‐NMR. The in vitro antifungal activities of all synthesized compounds were tested by the disk diffusion method against different phytopathogenic fungi. The results of the current experiment showed encouraging antibacterial effects. The key feature of the presented new protocol is the promising antifungal activity of the obtained products and the effective catalytic role of the IG‐BR cryogel. The research results revealed high product yields, environmental harmony, and the catalyst's recyclability as key points. Therefore, this study can offer an update on the development of high‐efficiency cryogels as an innovative class of catalysts among their multifield applications.

## Conflicts of Interest

The authors declare no conflicts of interest.

## Data Availability

The data that supports the findings of this study are available in the Supporting Information of this article.
